# Loading and Unloading Finishing Pigs: Effects of Bedding Types, Ramp Angle, and Bedding Moisture

**DOI:** 10.3390/ani5010013

**Published:** 2014-12-31

**Authors:** Arlene Garcia, John J. McGlone

**Affiliations:** Department of Animal and Food Sciences, Texas Tech University, Lubbock, TX 79409, USA; E-Mail: arlene.garcia@ttu.edu

**Keywords:** finishing pigs, ramp, slips, falls, vocalizations, animal welfare

## Abstract

**Simple Summary:**

Current guidelines suggest the use of ramps below 20 degrees to load and unload pigs; however, they do not suggest the use of any specific bedding. Bedding types (nothing, feed, sand, wood shavings, and hay) were tested with finishing pigs (70–120 kg) to determine which was most effective in reducing slips, falls, and vocalizations at three ramp angles, two moisture levels, over two seasons. Slips, falls, and vocalizations were summed to establish a scoring system for the types of beddings. Heart rate and the total time it took to load and unload pigs, increased as the slope increased. Bedding, bedding moisture, season, and ramp slope interacted to impact the total time it took for finishing pigs to load and unload the ramp. Selection of the best bedding depends on ramp slope, season, and wetness of bedding.

**Abstract:**

The use of non-slip surfaces during loading and unloading of finishing pigs plays an important role in animal welfare and economics of the pork industry. Currently, the guidelines available only suggest the use of ramps with a slope below 20 degrees to load and unload pigs. However, the total time it takes to load and unload animals and slips, falls, and vocalizations are a welfare concern. Three ramp angles (0, 10 or 20 degrees), five bedding materials (nothing, sand, feed, wood shavings or wheat straw hay), two moistures (dry or wet bedding, >50% moisture) over two seasons (>23.9 °C summer, <23.9 °C winter) were assessed for slips/falls/vocalizations (n = 2400 pig observations) and analyzed with a scoring system. The use of bedding during summer or winter played a role in the total time it took to load and unload the ramp (*p* < 0.05). Bedding, bedding moisture, season, and slope significantly interacted to impact the total time to load and unload finishing pigs (*p* < 0.05). Heart rate and the total time it took to load and unload the ramp increased as the slope of the ramp increased (*p* < 0.05). Heart rates were higher during the summer than winter, and summer heart rates increased as the slope increased (*p* < 0.05). The current study suggests that several factors should be considered in combination to identify the appropriate bedding for the specific occasion.

## 1. Introduction

Loading and unloading pigs for transportation plays an important role in animal welfare and economics of the pork industry. Pork quality and yield are greatly affected by pre-slaughter handling [1]. Loading and unloading of pigs is considered to be the most difficult part of the transport stage and can be a major stressor for pigs, compromising animal welfare. Pigs moved from grower-finisher environments to stunning at the abattoir that become non-ambulatory or die at any stage of the marketing process represent transport losses [2] and are a welfare concern. Transport losses present animal welfare, legal, and economic challenges for the US swine industry [2].Transport losses represent multimillion dollar losses for the pork industry, have become a swine well-being priority, and rules and regulations have been developed because of them [3]. Transport losses are multifactorial and involve people, pigs, facility design, management, transportation, slaughter facility, and environmental factors [2]. Additionally, transport losses may be influenced by factors such as genetics, carcass muscling, health status, structural soundness, body weight, nutrition, and conditions during transport [4]. The incidence of transport losses is estimated to be about 1% of all pigs marketed [5,6]. Steep loading ramps also compromise animal well-being, as they are commonly associated with injuries and prolapses [7,8]. For cattle, pigs, and sheep the maximum recommended angle for adjustable ramps is 25 degrees and 20 degrees for non-adjustable ramps [9]. Currently, the guidelines available only suggest the use of ramps below 20 degrees to load and unload pigs. However, these guidelines do not suggest the use of non-slip materials on the ramp floor.

Slipping is defined as a loss of balance without the body touching the floor, while falling is defined as a loss of balance with part of the body other than the legs in contact with the floor [10]. Slipping and falling can represent a welfare problem because they can cause stress and injuries to animals. Pot-belly and straight trailer designs are the most commonly used in the United States to transport pigs. The internal ramps of pot-belly trailers can compromise welfare due to slips and falls during loading and unloading [11] and possibly cause transport losses because of the higher number of the internal ramps the pigs are exposed to. A good transportation system should have well designed and carefully monitored facilities for loading and unloading, a holding area, and the veterinary care of animals [12]. It is also important to train employees on how to handle animals, using methods that are less stressful, and possibly even conduct weekly audits with a numerical scoring system to ensure that high welfare standards are maintained [12]. Reducing stress at loading and unloading could potentially reduce the number of dead and down pigs during transport.

Pigs may also refuse to load when it is either too cold or too bright outside, including baulking if the air is blowing in their faces [13]. Behavioral responses when loading pigs can indicate an animal’s aversion to a situation and can be characterized by freezing, not moving forward, backing up, running away, or vocalizing [14]. Social species such as pigs will vocalize excessively when caught or hurt [14]. The reactions to unpleasant stimulation may vary from animal to animal and from species to species [14,15] and can contribute to prolonged loading and unloading times.

Heart rate may also be used as a welfare assessment measure. Heart rate variability has been used in animal research to analyze changes in sympathovagal balance related to diseases, psychological and environmental stressors or individual characteristics such as temperament and coping strategies [16].

We recently reported the effects of ramp angle, bedding type, bedding moisture, and season on loading and unloading of weaned pigs [17]. The objective of this study was to investigate the effects of welfare of finishing pigs being loaded and unloaded at three ramp angles using different bedding material at different moisture levels over two seasons.

## 2. Experimental Section

Pigs were PIC USA genetics using the Camborough-22 sow line and the 280 boar line and weighed between 70 kg and 120 kg. All animals were fed a diet to meet or exceed NRC nutrient requirements. Feed and water were provided ad libitum. All animal procedures were approved by the Texas Tech University Animal Care and Use Committee.

Three ramp angles (0, 10 or, 20 degrees), five bedding materials nothing (N), sand (S), feed (F), wood/pine shavings (WS) or wheat straw (H), and two moistures (dry or wet bedding or floor) over two seasons (>23.9 °C summer, <23.9 °C winter) were assessed for slips/falls and vocalizations on finishing barrows and gilts. The study included 240 finishing pigs in a multifactorial design (5 beddings × 2 moistures × 3 slopes × 2 seasons = 60 treatments). Pigs were handled in units of four pigs per group. Five, 4-pig replications were evaluated per treatment. There were a total of 1200 pig observations (4 animals/treatment × 60 treatments × 5 replications). Since the number of required animals was high for finishing pigs, each group of four pigs was used to evaluate no more than 10 randomly-selected treatments out of the 60 possible treatments. Pigs that were injured, lame, or apparently sick were not used in the study.

The bedding material was either dry (greater than 80% dry matter with a target of 90% dry matter) or wet (less than 80% dry matter with a target of 90% wet matter). When nothing was used as a bedding, the entire ramp was wet or dry during each loading and unloading session. The bedding material varied in price. The cost for feed for a 45 kg bag was $18.00 (28 ± 2 kg were used for five replications, cost was approximately $12.00). The cost for sand for a 22.7 kg bag was $3.59 and three bags were used (65 ± 3 kg of sand were used for five replications, cost was approximately $10.77). The cost for wood shavings was $7.99 (10 ± 2 cubic feet of shavings were used for five replications, equivalent to one bale of shavings). The cost for hay varies depending on the season, and can range from $8.00–$11.85/bale in the area the study was conducted (24 ± 2 kg of hay were used for five replications).

Seasons were determined by outside air temperature. Temperatures were categorized into summer (>23.9 °C to <37.8 °C) and winter (>−6.7 °C to <23.9 °C). The average ambient temperature for winter was 8.7 °C and for summer it was 29.3 °C. Actual air temperature was used as a covariate within season in the statistical model, as well as air temperature effects. Temperature, humidity and wind speed outside the building were recorded every 5 min using a Kestrel^®^ 4500 (Nielsen-Kellerman, Boothwyn, PA, USA). All data was collected from 7 am to 5 pm.

Pigs had not been previously exposed to any of the bedding materials prior to the study. When bedding was used on the ramp, it covered all the floor surface of the ramp. The bedding was replaced after five replications of dry or wet treatments, but if bare parts of the ramp were exposed more bedding was added. When wood shavings and straw were used as bedding material, its depth was 9.5 mm which is equivalent to using one bale of wood shavings in a 1.3 m × 2.5 m ramp. Similarly, when feed (a non-pelleted combination of corn and soybean meal) and sand were used, 6.5 mm depth of bedding was used to cover the entire ramp floor surface.

Pigs were housed in groups of 10 in wire floored pens (2.1 m × 3.7 m). Pigs were not fasted prior to the study as occurs during regular transportation. Two pigs were randomly selected, isolated, and cornered with a sorting board, and heart rate monitors (Polar^®^ RS800CX) were placed around their chests to collect heart rate (beats per minute, BPM) information during loading and unloading. A total of four randomly selected pigs (including those with heart monitors) were removed from their home pen and walked a distance of 37.5 to 46.7 m inside the building with a 1.2 m wide aisle. The heart rate monitors were started during loading once the pigs reached the door, but prior to loading the ramp. When pigs were reluctant to move, a high pitch whistling sound was made, or a sorting board was used. The four pigs walked a distance of 4.6 m on the ramp with the random treatment on it and onto a trailer. The ramp also had cleats 0.3 m apart to prevent slips and falls. The ramp had a metallic chute, with a total length of 3.6 m and adjustable height. The chute was solid on the sides to 0.9 m high, then partially open above 0.9 m above the solid side. Pigs were moved the same distance irrespectively of ramp angle to get inside the trailer.

The trailer the pigs were loaded on remained stationary. After pigs were loaded onto the trailer they were moved past the adjustable back door and into a pen. The trailer pens were 2.1 × 2.4 m dimension. Pigs remained on the trailer 30 min. The heart rate monitors were stopped after the pigs loaded the trailer, but were kept on the pigs and restarted before they were unloaded. Pigs were unloaded from the trailer, moved down the ramp with the same treatment and returned to their home pen. Only two trained personnel were involved in moving the pigs and observing the video.

All the behavioral measures were recorded using digital Sony® camcorders DCR-SR85 (Sony, San Diego, CA, USA). A wide angle camcorder was fitted at the back of the trailer facing towards the exit door of the barn to record slips, falls and vocalizations as they loaded the ramp. Another digital camcorder was placed above the barn door to record slips, falls, and vocalizations as the pigs unloaded the ramp. Digital camcorders (Sony) were placed so that the first and last steps on and off the ramp were recorded in order to determine the total time to load and unload. The time of loading and unloading was determined by the first pig’s step onto the ramp and ended when the last pig stepped off the ramp onto the trailer (loading) or onto the aisle (unloading). The loading and unloading times were added to determine the total time. Video was downloaded and only two personnel were trained to recognize slips, falls, and vocalizations. The two trained personnel observed the video and analyzed it for slips, falls, and vocalizations. Animals that slipped and fell could be isolated from the others, animals that vocalized were not able to be isolated, but could be logged.

The sum of the slips, falls, and vocalizations were recorded as a score in part because the data set for any one measure contained many zero values. Treatments were then given a score based on the number of times the animals slipped, fell, and vocalized. As the slips, falls, and vocalizations increased, the score increased. Scores ranged from 0 to 91. Lower scores meant a lower number of slips, falls, and vocalizations which was considered better than high scores. Slips were defined as when one foot missed a step but the pig caught itself; falling was considered an imbalance of the pig’s body with some part of the body physically touching the floor; vocalizations were any squeals produced by the pigs other than grunts.

### Statistical Analysis

The study used a Complete Randomized Design with five repetitions per treatment for a total of 60 treatments. A general linear model was used and all the data were analyzed using analysis of variance procedures in SAS 9.3 General Linear Models procedure (SAS, 2010 SAS Inst., Inc., Cary, NC, USA). A predicted Student’s T test was run within SAS. The statistical model included the effects of bedding, slope, wet/dry, season, heart rate, all possible interactions, and temperature and wind as a covariate. All data were tested for homogenous variances and normal distributions. The experimental unit was a group of four pigs.

## 3. Results and Discussion

Bedding types (nothing, feed, sand, wood shavings, and hay) were used on a ramp to determine which was more effective in preventing slips, falls, and vocalizations at different angles (0, 10, 20), moisture levels (wet or dry) and seasons (summer or winter). The score combined each of the measures (slips, falls, and vocalizations). Because so many observations were zero (example: there were no slips, falls or vocalizations at zero degree slope), the score may be the most robust measure. A combined view of score and total time (TTime) to load and unload gives the best overall view of the results. However, score was not significantly affected by treatment for finishing pigs as previously reported in weaned pigs [17].

Heart rates (BPM) were recorded for each pig and the average heart rates for each treatment were established. Main effects are summarized first followed by interactions.

### 3.1. Total Time: Slope Effect

**Figure 1:**
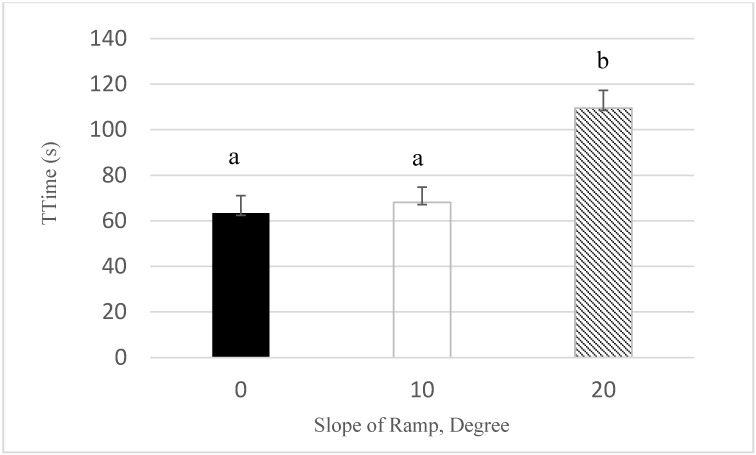
Least Squares means for total time (TTime, s) it took to load and unload pigs on a ramp with slopes of 0, 10, 20 (*p* < 0.01). N = 100 observation of four pigs each. Superscripts without a common letter are different at *p* < 0.05.

Slope played an important role in the amount of time it took to load and unload finishing pigs on the ramp. As the slope increased the time it took to load and unload increased (Figure 1). In the current study the slope and the use of bedding did not significantly affect scores, but did affect loading and unloading times as well as heart rates. The delay in loading and unloading due to unmanageable pigs may be frustrating to the handler, and even small amounts of threatening behaviors by humans can produce a chronic stress response in pigs [18]. Aggressive handling, including the use of electric prods, produce a major metabolic response that results in an increase in body temperature, decreased blood pH, and a high incidence of fatigued pigs [19]. The amount of time spent loading and unloading is important in the swine industry since loading pigs is considered the most critical part of the transport stage.

Previous studies have documented increases in unloading times due to slopes greater than 20 degrees [11], an increase in baulking of pigs [20] and increased difficulty in handling of pigs [21]. Furthermore, at loading, high live weight pigs make a considerable physical effort to go up ramps [22]. Thus, the current results corroborate previous negative effects of the use of steep ramp slopes.

### 3.2. Total Time: Interaction for Bedding by Season

**Figure 2:**
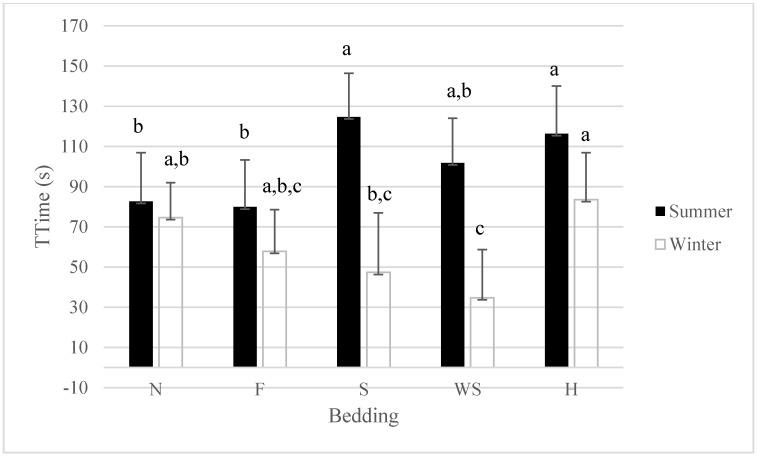
Least Squares means for total time for bedding x season interaction (*p* < 0.05). N = 30 observations. Bedding abbreviations N = nothing, F = feed, S = sand, WS = wood shavings, H = hay. Superscripts without a common letter for total time within season are different at *p* < 0.05.

Season (whether summer or winter) affected the amount of time it took the pigs to load and unload the ramp. There was a significant interaction for season and bedding (*p* < 0.05). The time it took to load and unload finishing pigs during the summer was highest for sand and hay, respectively 125 ± 21.7 s and 116 ± 23.7 s (Figure 2). It took a shorter amount of time for finishing pigs to load and unload when feed or when nothing was placed on the ramp during the summer, respectively 80 ± 23.4 s and 83 ± 24.3 s. Feed was used as a bedding because it is sometimes used by people trying to load pigs at the farm when other bedding is not available. According to this study, the use of feed is an effective method to reduce loading and unloading times in the summer, but not using any bedding on the ramp was also acceptable in the summer. During the winter months, the use of wood shavings, sand, and feed had the lowest loading and unloading times, respectively, 35 ± 24.1 s, 47 ± 29.6 s, and 58 ±20.1 s. Differences in the total time it took to load and unload with different beddings may have been due to preferences in the smell and consistency of the beddings. For example, feed placed on the ramp was the same type of feed the pigs were fed on a daily basis and the smell may not have been novel to them, hence not attracting them to stay longer smelling and investigating it. The other beddings were novel and this may be the reason total handling times increased with their use. The novelty effect plus the fact that hay was edible may have been another reason total loading and unloading times increased with its use. Sand and wood shavings were not significantly different than feed, they were novel, but not edible (compared to hay). Overall, the use of bedding on ramps and season effect on loading and unloading times has been poorly documented and makes it difficult to compare current results to previous findings.

### 3.3. Total Time: Interaction for Bedding, Moisture, Season, and Slope

The total time it took to load and unload the pigs varied with the type of bedding, moisture, season, and slope. The longest time for loading and unloading was during the summer, with a slope of 20 degrees, using dry sand as a bedding, with a total time of 302.8 ± 34.7 s. There was a significant reduction (*p* < 0.05) in the amount of time it took to load and unload the pigs using dry sand as a bedding when the slope was 0 degrees or 10 degrees (respectively 58 ± 33 s and 86 ± 36.2 s). Loading and unloading times for sand in the winter were not significantly different, regardless of moisture status or ramp angle. The shortest time to load and unload was with a 0 degree slope using dry wood shavings as a bedding in the winter (25 ± 33.6 s). There was no significant increase in total time with its use, regardless of slope (25 ± 49.1 with 10 s degree slope and 50 ± 34.5 s with a 20 degree slope) and moisture. However, when wood shavings was used in the summer, loading and unloading times increased with its use (65 ± 37 s lowest loading and unloading times; 180 ± 32.9 longest amount of loading and unloading time). The use of dry hay increased total times significantly from a 0 degree slope to a 20 degree slope during the summer. There were not any significant differences in loading and unloading times when used wet, regardless of slope during the summer. During the winter there was a significant difference in loading and unloading times with the use of dry hay at 10 and 20 degrees (respectively, 34.4 ± 40.5 s and 183.2 ± 51.4 s). No significant differences in loading and unloading times were seen with the use of wet hay during the winter, regardless of slope. It must be taken into account that pigs are socially investigative (investigate con-specifics) or non-socially investigative (investigate the environment) [23]. Either the smell or the consistency of the bedding in the current study seemed to cause the pigs to increase non-socially investigative behaviors. In commercial settings where pigs are typically loaded and unloaded quickly, slow loading and unloading times caused by increased exploration could potentially create additional problems. Some of the delays in loading or unloading may not directly be caused by bedding. Causes for delays in loading and unloading can include aversion to shadows, noise, either too cold or too bright outside [13,24], current injuries the animals may have that prevent them from loading rapidly, novelty of objects, interaction with humans, and other undermined causations. Hence, aversive situations can increase loading and unloading times because they are characterized by freezing, not moving forward, backing up, running away, or vocalizing [25]. Good methods to decrease stress and loading times may also include having pens positioned next to the loading area, not mixing unfamiliar pigs, regular handling of pigs when they are young [25,26], and moving them in small groups, rather than in big groups or individually when they are being loaded [24].

### 3.4. Heart Rate: Interaction for Slope by Season

**Figure 3:**
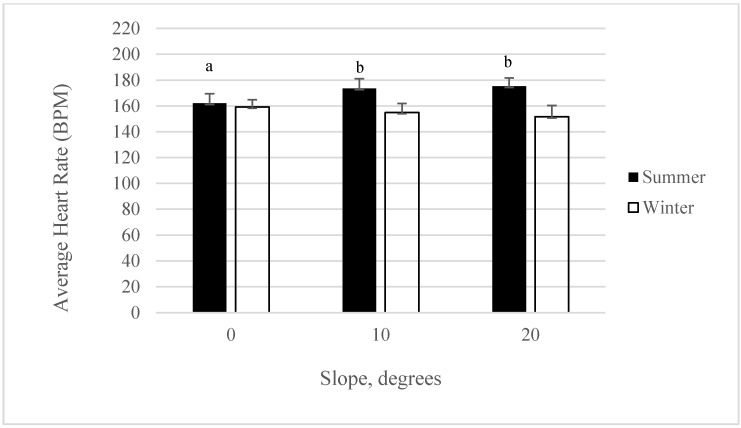
Least Squares means for average heart rate at different slopes during summer or winter season (*p* < 0.05). Superscripts without a common letter are different at *p* < 0.05 within season.

Heart rates in finishing pigs increased as the slope increased and were observed to be higher during the summer than during the winter (*p* < 0.05). Heart rates at a 0 degree slope during the summer were significantly lower than at 10 and 20 degree slopes (*p* < 0.05*)* (Figure 3). Heart rates did not differ during the winter, regardless of slope. The increase in heart rates during the summer may also be attributed to heat stress and not solely just the bedding and slope of the ramp. Both high environmental temperatures in summer and temperature fluctuations affect the animal’s ability to maintain body temperature which results in stress [27]. Heart rate is an important measure commonly used to evaluate animal welfare during stimulated handling and transportation [28]. Heart rate is used as a sign of autonomic response to stress and welfare of animals during exposure to stressors [29] and may also be related to body weight [28]. Pigs may perceive environmental aspects as aversive, and it has been suggested that noise may also be a disturbing factor that may increase heart rate [30].The number of studies looking at heart rate and its association to bedding and season have not been widely documented. However, a direct positive correlation between heart rate during loading and ramp slope have been reported [24,25,26,27,28,29,30,31]. Alternative methods to loading, such as the use of tailgate lifts have been reported to reduce heart rate in comparison to ramp loading [32].

### 3.5. Heart Rate: Interaction for Bedding by Season

**Figure 4:**
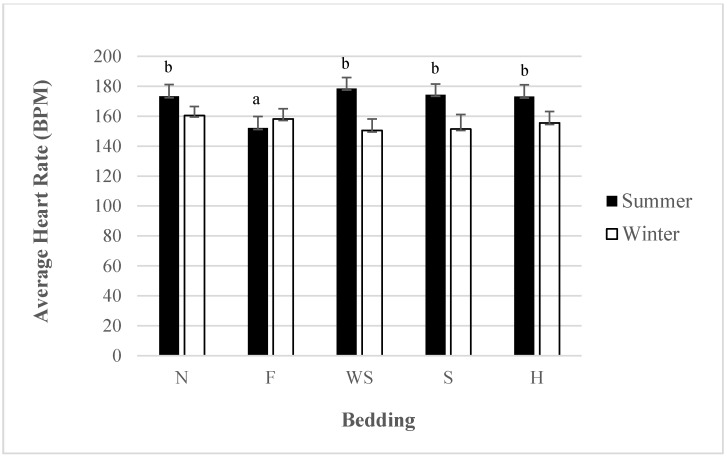
Least Squares means for average heart rate for different beddings during summer or winter season (*p* < 0.01). Bedding abbreviations N = nothing, F = feed, S = sand, WS = wood shavings, H = hay. Average heart rate within season. Superscripts without a common letter are different at *p* < 0.05 within bedding.

The use of bedding on the ramp significantly affected finishing pig heart rates in the summer (*p* < 0.01) but not in the winter. Heart rates were lower for feed than they were for other beddings during the summer (Figure 4). All other beddings did not differ in their effect on heart rates during the summer. The use of feed also had lower loading and unloading times, possibly suggesting that familiarity with a bedding may reduce both heart rates and loading and unloading times. Additionally, there were not any differences in heart rates based on the bedding used during the winter.

### 3.6. Heart Rate: Interaction for Bedding by Slope

**Figure 5:**
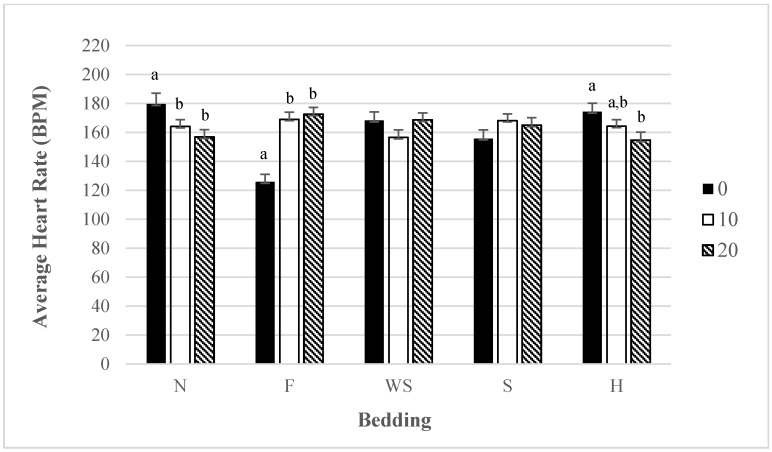
Least Squares means for average heart rate for different beddings 0, 10, and 20 degree slopes (*p* < 0.01). Beddings abbreviated by N = nothing, F = feed, S = sand, WS = wood shavings, H = hay. Superscripts without a common letter are different at *p* < 0.05 within bedding.

The use of bedding at different slopes either significantly reduced heart rates or increased them (*p* < 0.01). Using nothing at a 0 degree slope had higher heart rates than at 10 and 20 degree slopes, therefore heart rates decreases as the slope increased (Figure 5). This can possibly be attributed to the speed at which the finishing pigs went up the ramp. At a 0 degree slope, finishing pigs tended to run up the ramp when there was no bedding. Since the 0 degree slope is similar to being in the pen or walking down the aisle, the pigs navigated through the ramp faster. When the slope increased they slowed down their pace of loading, pigs may have been trying to maintain their equilibrium. The use of hay as a bedding had similar results as using nothing. Heart rates were higher at a 0 degree slope and they decreased as the slope increased. When feed was used on the ramp heart rates increased as the slope increased. Heart rates were significantly higher at 10 and 20 degree slopes overall during the summer compared to a 0 degree sloped ramp (level floor). An increase in heart rates has been documented to increase as the slope increases from 0 to 21 degree slopes [21].

### 3.7. Heart Rate: Interaction for Bedding, Season, and Slope

The use of bedding during either summer or winter at 0, 10, and 20 degree slopes had a significant effect on heart rate (*p* < 0.05). Using nothing on the ramp during the summer season at a 0 degree slope had a higher heart rate than at a 20 degree slope (Table 1). The use of hay during the summer season had similar results as using nothing. Heart rates were higher at a 0 degree slope than with a 20 degree slope. The use of feed during the summer had a linear increase in heart rate, which is more typical of what should happen when pigs are loading and unloading the ramp. Heart rates during the summer were more elevated than they were during the winter.

**Table 1:** Least Squares means for heart rate (BPM) during loading and unloading in response to the three-way interaction of bedding, season, and slope (*p* < 0.05).

Bedding	Season	Slope, (°)	Avg HR (BPM)	SE
**Nothing**	Summer	0	184 ^a^	11.78
**Nothing**	Summer	10	172 ^a,b^	9.09
**Nothing**	Summer	20	164 ^b^	8.35
**Nothing**	Winter	0	175	9.16
**Nothing**	Winter	10	157	7.32
**Nothing**	Winter	20	150	10.91
**Feed**	Summer	0	103 ^a^	9.36
**Feed**	Summer	10	174 ^b^	9.87
**Feed**	Summer	20	180 ^b^	9.04
**Feed**	Winter	0	147	10.71
**Feed**	Winter	10	162	8.46
**Feed**	Winter	20	165	7.27
**Sand**	Summer	0	169	8.92
**Sand**	Summer	10	176	9.30
**Sand**	Summer	20	178	9.21
**Sand**	Winter	0	143	12.68
**Sand**	Winter	10	160	9.32
**Sand**	Winter	20	152	11.87
**Shavings**	Summer	0	178	11.11
**Shavings**	Summer	10	175	8.96
**Shavings**	Summer	20	183	8.20
**Shavings**	Winter	0	159	7.45
**Shavings**	Winter	10	138	11.80
**Shavings**	Winter	20	154	9.58
**Hay**	Summer	0	176	8.88
**Hay**	Summer	10	172	10.26
**Hay**	Summer	20	172	9.17
**Hay**	Winter	0	172 ^a^	7.54
**Hay**	Winter	10	157 ^a^	9.99

Superscripts without a common letter are different at *p* < 0.05 within bedding.

## 4. Conclusions

To our knowledge, the type of bedding to be used on ramps to reduce slips, falls, and vocalizations in finishing pigs during loading and unloading has not been evaluated. The use of some type of bedding when loading and unloading finishing pigs on a ramp is usually beneficial in reducing the total time it takes for the animals to load and unload. During the summer, any bedding aside from hay, even nothing at all can help reduce the total time it takes to load and unload. During winter, the use of wood shavings, feed, sand reduced total loading and unloading times compared to not using any bedding or using hay.

During the summer, reducing slopes will help reduce heart rates. The use of feed on the ramp reduced heart rates significantly compared to other beddings during the summer, especially if the slope was at 0 degrees. However, the cost of bedding may outweigh its benefits. Overall, it seems more economical to use wood shavings, and it also works as an effective bedding.

Slip-resistance of floors merits further study. Furthermore, slip-resistance of floors should be studied in combination with characteristics of flooring such as abrasion, surface profile, and hardness, to avoid injuries to pigs [33]. It is also important to understand that pigs can adapt to fouled floor conditions by reducing their walking speed and stride length, but it is not sufficient to ensure walking safety [34]. In addition, the use of hydraulic lifts or modular systems may be less physically demanding on animals [35] and also merits further study.

Overall, several factors should be considered in combination to identify the appropriate bedding for the specific occasion in order to address financial losses due to pre-slaughter handling and good animal welfare.
